# Involvement of Src in the Adaptation of Cancer Cells under Microenvironmental Stresses

**DOI:** 10.1155/2012/483796

**Published:** 2012-09-03

**Authors:** A. K. M. Mahbub Hasan, Takashi Ijiri, Ken-ichi Sato

**Affiliations:** ^1^Laboratory of Cell Signaling and Development, Department of Molecular Biosciences, Faculty of Life Sciences, Kyoto Sangyo University, Kyoto 603-8555, Japan; ^2^Laboratory of Gene Biology, Department of Biochemistry and Molecular Biology, University of Dhaka, Dhaka 1000, Bangladesh

## Abstract

Protein-tyrosine phosphorylation, which is catalyzed by protein-tyrosine kinase (PTK), plays a pivotal role in a variety of cellular functions related to health and disease. The discovery of the viral oncogene Src (v-Src) and its cellular nontransforming counterpart (c-Src), as the first example of PTK, has opened a window to study the relationship between protein-tyrosine phosphorylation and the biology and medicine of cancer. In this paper, we focus on the roles played by Src and other PTKs in cancer cell-specific behavior, that is, evasion of apoptosis or cell death under stressful extracellular and/or intracellular microenvironments (i.e., hypoxia, anoikis, hypoglycemia, and serum deprivation).

## 1. Introduction

It is believed that the ancient Greek physician Hippocrates (ca. 460 B.C.–ca. 370 B.C.), the father of medicine, was the first to use the word “cancer” in this context. Although phenomena reflecting the formation of malignant tumor had already been described much earlier, Hippocrates was the first to use the Greek word “carcinos” (in English and Latin, “cancer”), based on the word for crab, which he thought resembled the cut surface of a malignant tumor [1]. Long after this incident, the biology and medicine of cancer reached the age of modern science in the mid 18th century with findings and reports that some cases of cancer may be associated with the patient's lifestyle and/or job (e.g., nose, scrotum) [2, 3]. Currently, cancer is known as one of the most critical and fatal diseases in humans, especially in those living in areas with relatively high longevity. Thus, in general, cancer is recognized as having been relatively rare during the earlier average human lifetime. Nevertheless, overall, demands for understanding, preventing, and curing cancer are growing; therefore, the biology and medicine of cancer are of particular importance in science. 

 Why and how is cancer rare? Why and how does cancer arise and develop? Why and how is cancer fatal? Many fundamental questions arise from the study of cancer. Against this background, an extensive number of studies have been conducted in recent decades using many approaches including animal models, bioinformatics, and cellular and molecular biology techniques. In 2000, Hanahan and Weinberg, a pioneering scientist in the cancer biology field, proposed that the development of most cancer cells is the result of a manifestation of six essential alterations in cell physiology that collectively dictate malignant growth: self-sufficiency in growth signals, insensitivity to growth-inhibitory signals, evasion of programmed cell death or apoptosis, limitless replicative potential, sustained angiogenesis, and tissue invasion and metastasis [4]. These altered functions are based on the occurrence of critical mutations in one or more cancer-related genes (i.e., protooncogenes and/or tumor suppressor genes), as well as other cancer-promoting, nongenomic mechanisms involving epigenetically altered expression of certain genes and a number of environmental factors that could affect intracellular signaling events and/or metabolic systems. Under these circumstances, current trends in the biology of cancer deal extensively with the molecular details of how each type of human cancer cell arises, develops, and maintains its cancerous features as described previously, by which it shows aggressive and pathological behavior in the patients with such lesions.

 A century ago, Rous discovered a virus named Rous sarcoma virus (RSV) that has cell-transforming activity [5, 6] and carries a viral Src (*v-src*) oncogene [7–10]. *v-src* gene originated from a cellular progenitor termed protooncogene cellular Src (*c-src*) or Src; they share a conserved sequence that just differs by several point mutations throughout the gene and deletion mutations in the C-terminal region [10–13]. *c-src *is one of the oldest protooncogenes, discovered in 1976 in the vertebrate genome [13]; this outstanding discovery led to the Nobel Prize for Medicine and Physiology being awarded to Bishop and Varmus in 1989. The protein product of this gene is nonreceptor protein tyrosine kinase (PTK), Src, which principally attaches to the inner plasma membrane and associates with many kinds of cellular proteins that include receptor tyrosine kinases, G-protein-coupled receptors, steroid receptors, and signal transducers and activators of transcription (STAT) [14]. There are eleven identified Src family kinases (SFKs): Src, Fyn, Yes, Blk, Yrk, Fgr, Hck, Lck, Lyn, Frk (also known as Rak), and Srm. Src or SFK study has led to new insights into the role of tyrosine phosphorylation in cell physiology and functions (for review, see [15–19]). The diverse functions of Src involve the initiation of fertilization-mediated development and regulation of normal cell growth, survival, proliferation, differentiation, adhesion to matrix and a wide range of molecular signaling networks. Src protein is composed of four Src homology (SH) domains, a unique N-terminal domain, a linker region and negative-regulatory tyrosine residue (chicken Tyr527; human Tyr530)-containing C-terminal tail [17–22].

 Among the four SH domains, SH1 is the kinase domain that contains the autophosphorylation site required for full activity (chicken Tyr416; human Tyr419), SH2 interacts with the negative-regulatory Tyr527 (chicken) or Tyr530 (human), SH3 promotes intramolecular contact with the kinase domain for inactivation, and SH4 contains the myristoylation site that is important for lipid bilayer membrane localization. The functions of the unique N-terminal domain are not well understood, but mutation in this region seems to reduce the transforming potential of v-Src [21]. The SH2 and SH3 domains and C-terminal tail are involved in the negative regulation of Src. A linker region is present between SH2 and SH1 domains and is involved in intramolecular binding with the SH3 domain. Phosphorylated C-terminal tyrosine residue can bind to the SH2 domain. These interactions along with the interaction between the kinase domain and the SH3 domain cause the Src molecule to adopt a closed conformation that makes it unavailable for its substrate [23]. Dephosphorylation at the C-terminal tyrosine residue by tyrosine phosphatase or the Src-interacting molecules that break the closed conformation without C-terminal tyrosine dephosphorylation converts the Src molecules into an open active state. Consistently, v-Src is constitutively active because it lacks the C-terminal negative-regulatory tyrosine residue.

 Apoptosis is critical for maintaining the appropriate cell number in tissues and organs. Apoptosis is somehow escaped in transformed or cancer cells, leading to their immortality by a mechanism called anti-apoptosis. Src plays roles in several types of cancer cells, such as breast cancer [24] and an astrocytoma cell line [25], to provide them with an anti-apoptotic character, and even acts in vascular endothelial growth factor (VEGF)-induced endothelial cell anti-apoptosis [26]. Mutations of several other molecules, for example Raf, Ras, and STAT, also contribute to anti-apoptosis, abnormal proliferation, angiogenesis, and invasion of several types of cancer cells, such as in melanoma and gliomas [27–30]. How normal cells are transformed into cancer cells and progress to invasive cancers and then to metastatic mode and their relationships with Src are very interesting issues. Src has the potential to be altered in a fashion that allows it to play a role in cancer progression. When cells are transformed, they lose molecular controls and subcellular structures and ultimately alter their cell-cell and cell-matrix interactions and become motile and invasive; Src plays a central role in this process [4, 31, 32]. Increased Src kinase activity is associated with advanced-stage tumors that readily metastasize to distant organs [33–36]. The activation of Src in human cancers may occur through a variety of mechanisms that include domain interaction between molecules and/or structural remodeling in response to multiple activators or upstream kinases and tyrosine phosphatases. Overexpression of Csk, a negative regulator of Src, suppresses metastasis in mouse model experiments, demonstrating the importance of Src activity in metastasis [37]. The involvement of Src activity has been studied in several carcinomas, including colorectal, hepatocellular, pancreatic, gastric, esophageal, breast, ovarian, lung, and prostate carcinomas [38–42]. In some cases, for example, hepatocellular and colon carcinomas, very high Src activity and low expression of Csk were observed [43–45]. Recently, the oncoprotein Src has been focused on as a molecular target for cancer therapy. Several Src inhibitors that are highly specific and stable *in vivo* have been extensively studied, and attempts are now underway to utilize them in human cancer treatment because blocking of Src activation may inhibit several signaling pathways involved in tumor progression [46–50]. However, successful targeting of Src in a clinical setting remains a challenge, and Src inhibitors have only recently started to move through clinical development.

 When a population of cancer cells arises, surrounded by normal cells and tissues, these cancer cells will suffer from various kinds of environmental stress, such as low oxygen pressure (i.e., hypoxia), lack of cell-cell contact (low confluence) and insufficient support by the extracellular matrix (possibly leading to anoikis), and shortage or complete lack of nutrients (e.g., hypoglycemia) and growth factors (e.g., low serum). These microenvironmental stresses could act as selective pressures or death-promoting (e.g., pro-apoptotic) signals for cancer cells, so that only those that successfully adapt to them can continue their malignant growth. Some cancer cells with relatively high malignant potential overcome this situation by triggering altered gene expression (e.g., upregulation of hypoxia-inducible genes) and signal transduction for angiogenesis (e.g., expression of vascular endothelial growth factor) [51, 52]. In this paper, we discuss how these cancer-specific adaptations to microenvironmental stresses are managed with a focus on the roles of Src and other PTKs and how this knowledge could contribute to future progress in this research field.

## 2. Hypoxia

Tumor blood microvessels arising from neovascularization are structurally and physiologically different from normal blood vessels. Tumor blood vessels are highly irregular (displaced and compressed), tortuous, have arteriovenous shunts and blind ends, are leaky, lack smooth muscle or enervation, and have incomplete endothelial linings and basement membranes that often result in sluggish, highly abnormal blood flow [53–55]. Because of unrestrained growth, tumor cells are forced away from vessels beyond the effective diffusion distance of oxygen in respiring tissue and suffer from hypoxia, a lack of oxygen that is the result of an imbalance in oxygen supply and demand [56]. In hypoxic regions, the partial pressure of O_2_ (pO_2_) levels are chronically low, and, in addition, owing to intermittent blood flow acute hypoxia is produced followed by reoxygenation [52, 55, 57].

 Hypoxia is deleterious to cancer and normal cells, but the conditions that are prevalent in solid tumors are believed to exert selective pressure for cancer cells to adapt and survive. During growth and metastatic progression, tumor cells encounter several kinds of microenvironmental stresses; the most critical of which is hypoxia [58]. Oxygen limitation is central in controlling neovascularization, glucose metabolism, tumor survival and spread. This pleiotropic action is orchestrated by hypoxia-inducible factor-1 (HIF-1), which is a master heterodimeric transcriptional factor mediating a wide range of physiological and cellular mechanisms; this action can be termed an angiogenic switch to overcome the limited supply of oxygen and nutrients in expanding neoplasia [58–61]. During hypoxia, HIF-1*α* is stabilized and translocates to the nucleus where it forms a heterodimer with HIF-1*β* [62]. This HIF-1 complex interacts with hypoxia-responsive elements and regulates the expression of molecules such as the major pH-regulating enzyme carbonic anhydrase IX, which allows metabolic adaptation in the cell [63, 64].

 There is considerable interest in understanding the molecular mechanisms involving several oncogenes and oncogenic molecules that enhance tumor angiogenesis and malignant progression under hypoxia. Tumor hypoxia promotes metastasis via the upregulation of many genes including VEGF, c-Met, and C-X-C chemokine receptor type 4 (also known as CD184), which are integral to metastatic tumor progression [65–69]. The hypoxic microenvironments are also associated with alterations in signaling proteins including Src, STAT3, phosphoinositide 3-kinase (PI3K)/Akt, extracellular signal-regulated kinase (Erk, also known as mitogen-activated protein kinase), and glycogen synthase kinase 3*β* (GSK3*β*), which are generally considered to be prosurvival (anti-apoptotic) and are commonly activated in cancer. It was shown that HIF-1*α* expression requires PI3K activity and is correlated with Akt phosphorylation in invasive breast carcinomas [70]. Akt can augment HIF-1*α* expression by increasing its translation under both hypoxic and normoxic conditions [71]. Hypoxia-induced activation of PI3K/Akt occurs at an early stage, but prolonged hypoxia inactivates Akt and activates GSK3*β*, which then downregulates the HIF-1 activity through downregulation of HIF-1*α* accumulation [72]. Erk is also needed for hypoxia-induced HIF-1 transactivation activity because HIF-1*α* is phosphorylated in hypoxia by an Erk-dependent pathway [73]. Recently, it was suggested that Src activation might play a prominent role in the response to hypoxia to promote human cancer cell survival, progression, and metastasis. Src-nuclear factor kappa B (NF*κ*B) was shown to contribute to the survival of cells during hypoxia as Src inhibition causes hypoxia-induced cell death [74]. In both pancreatic and prostate carcinoma cell lines, it was shown that artificial hypoxia (by cobalt chloride)-induced VEGF expression required Src activation and resulted in increased steady-state levels of HIF-1*α* and increased phosphorylation of STAT3. STAT3 and HIF-1*α* bind simultaneously to the VEGF promoter for maximum transcription of VEGF mRNA following hypoxia [75]. STAT3 activity is responsive to acute hypoxia, whereas the signaling from Src to focal adhesion kinase (FAK) is associated with chronically hypoxic regions [76].

 Hypoxia/reoxygenation (H/R) regulates Lck (a member of SFKs)-dependent activation of NF*κ*B (nuclear factor *κ*B) and modulates the expression of downstream genes that are involved in cell migration in human breast cancer cells. H/R-activated Lck mediates NF*κ*B activation, urokinase-type plasminogen activator secretion, and cell motility through tyrosine phosphorylation of I*κ*B*α* (inhibitor of nuclear factor kappa B, alpha) [77]. It has been documented that a nonreceptor and non-Src family PTK, Syk, is commonly expressed in normal human breast tissue and breast tumor [78]. Syk and Lck together regulate H/R-induced breast cancer progression [79], but the molecular mechanism of Syk phosphorylation and its subsequent interaction with Lck leading to downstream signaling events are not well defined. H/R enhances the production of reactive oxygen species that cause the inhibitory oxidation of protein tyrosine phosphatase (PTP), a major regulator of tyrosine kinase signaling [80]. Hypoxia has also been shown to upregulate lysyl oxidase (LOX) expression via HIF-1 binding to hypoxia-responsive elements in the LOX promoter, leading to enhanced invasion in metastatic breast cancer [81, 82]. H/R condition stimulates the LOX-dependent FAK/Src activity, which facilitates breast cancer cell migration through a mechanism mediated by hydrogen peroxide, a by-product of LOX activity [82–84]. Thus, the key molecules, for example, Src, FAK, PI3K, and LOX, involved in the anti-apoptosis process of cancer cells could be good therapeutic targets for preventing and treating metastases. However, most of the data have been obtained from *in vitro* experiments, but the microenvironment in the hypoxic tumor is likely to be more complicated due to the existence of pO_2_ gradients, temporal fluctuations in pO_2_, tumor/stroma interactions, and the additional effects of nutrient availability and acidosis.

## 3. Matrix Deprivation

Cells are held tightly in a highly structured order with each other (cell-cell contact) and with their surrounding extracellular matrix (ECM) (cell-ECM contact) for their mutual benefit. These associations are principally regulated by the cellular membrane protein integrins that execute signals through cytosolic molecules for their survival when they are attached. If cells are detached or their cell-cell contact or cell-ECM contact is severed, they die by a particular type of apoptosis, “anoikis” (from the Greek word for “homelessness”), which was first described for epithelial cells but later shown also to take place in cells of nonepithelial origin [85–87]. Detachment-induced anoikis is physiologically significant for normal cellular growth and turnover. However, cancer cells are insensitive to this death process. Resistance to anoikis is very important for cancer cells because they can survive after detachment and undergo metastasis to distant organs. The molecular mechanisms by which cancer cells escape anoikis are not clearly understood, but recent study has shed some light on this issue. Integrins, transmembrane heterodimers, and two principal nonreceptor tyrosine kinases, FAK and Src, play a central role in resisting anoikis [88, 89]. In general, anoikis resistance (or anti-apoptotic signal) involves a conformational change of integrin, which recruits autophosphorylated FAK at Tyr397. Src interacts with the activated FAK through the Src's SH2 domain, resulting in the activation of Src by tyrosine autophosphorylation. Activated Src phosphorylates FAK (Tyr861 and Tyr925) to enhance its activity further. This activated FAK/Src complex conveys the signal for anoikis resistance through the PI3K/Akt survival pathway [86, 90, 91]. Thus, the downregulation and loss of FAK/Src association are involved in anoikis sensitivity.

 The role of integrins, FAK, Src, and PI3K/Akt differs depending on the cancer cell types. Anchorage-independent growth and survival of pancreatic cancer cells require the recruitment of Src to the *α*v*β*3 cytoplasmic tail of integrins, leading to Src activation and Crk-associated substrate (also called CAS) phosphorylation but this is independent of FAK activity [92, 93]. Similarly, anoikis resistance is maintained in osteosarcoma cells through Src-dependent activation of the PI3K/Akt pathway in a manner independent of FAK activity [94]. Platelet-derived growth factor receptor (PDGFR), not FAK or epidermal growth factor receptor/kinase (EGFR), acts as the upstream PTK responsible for the detachment-induced Src activation; in addition, Pyk2 (a nonreceptor and a non-Src family PTK), rather than PI3K/Akt or Erk, acts as the key downstream effector of Src in mediating the cell survival signals of lung tumor cells [95, 96]. Intestinal epithelial cancer cells commonly display EGFR-mediated sustained activation of Src interacting with FAK and consequent MEK/Erk activation to resist anoikis [97]. The involvement of FAK/Src activity through the PI3K/Akt pathway in decreased sensitivity to anoikis has been described for human lung cancer [98, 99] and colon tumor cell lines [100, 101]. This differential regulation of anoikis-resistance/anti-apoptosis signaling pathway might develop in different tissues during differentiation and/or at the time of tumor growth and invasion. Thus, at present, it may be concluded that the FAK/Src complex is a potential target to treat the tumors and to stop their invasion.

## 4. Glucose Deprivation

It was more than 80 years ago that Warburg made an observation that transformed cells employ aerobic glycolysis for their energy production, rather than electron transport chain activity in mitochondria [102]. Several oncogenes including Akt, Ras, and Src activate the Warburg effect by increasing glucose uptake, transcription of enzymes involved in glucose metabolism, and aerobic glycolysis itself [103, 104]. The mechanism by which the Warburg effect is manifested in these transformed cells is still unknown. However, studies using Src-transformed fibroblasts demonstrated that Src induces elevated expression of HIF-1*α* in mRNA and protein level under normoxia [105, 106]. Pancreatic and prostate cancers have also been shown to involve Src-dependent induction of VEGF through the actions of HIF-1*α* [75]. These features seem to be peculiar to the transformed cells because another report has shown that, in Hep3B cells, in which Src activity has been manipulated, Src activity is not involved in the upregulation of HIF-1*α* and other HIF-1*α*-dependent phenomena [107]. HIF-1*α* is a transcriptional regulator, whose up-regulation normally occurs under hypoxia and activates glycolysis, erythropoiesis, and angiogenesis [52, 106]. Therefore, the up-regulation of HIF-1*α* by an Src (or other oncogenic factor)-dependent mechanism may explain at least to some extent how the transformed cells acquire the ability to develop the Warburg effect. Other oncogenic factors that induce the expression of the mRNA and/or protein of HIF-1*α* include ligands for some receptors/PTKs (e.g., EGF) [108], ErbB2/PTK [109], Ras [110], and STAT3 [111, 112]. Consequently, it is expected that glucose deprivation, or hypoglycemia, would lead to a failure of the Warburg effect to occur and affect cancer cell survival and proliferation.

 In breast carcinoma MCF-7/ADR cells, glucose deprivation causes an immediate increase in tyrosine phosphorylation and activates Lyn, but not Abl, Fyn, or Lck [113]. It has been shown that the hypoglycemia-induced Lyn activation is responsible for the subsequent activation of c-Jun N-terminal kinase (JNK), which then phosphorylates and activates a transcription factor c-Jun. Under these conditions, the JNK/c-Jun pathway increases in terms of total glutathione, cysteine, *γ*-glutamylcysteine, and immunoreactive proteins, corresponding to the catalytic as well as regulatory subunits of *γ*-glutamylcysteine synthetase. This suggests that the synthesis of glutathione is increased as an adaptive response. These metabolically produced substances culminate in manifestation of oxidative stress in the cells and lead to cell death [114]. Thus, the results suggest that a certain kind of Src family PTK (not necessarily Src) signaling can contribute to the hypoglycemia-induced death of cancer cells. Such modulated sensitivity to cell death or apoptosis under glucose-deprived conditions is also seen in the multipotential hematopoietic cell line 32D expressing v-Src, v-Ras, and v-Abl [115]. In this cell line, these oncogenes enhance apoptosis induced by hypoglycemia but attenuate apoptosis in the absence of IL-3. Interestingly, Bcr-Abl tyrosine kinase, an oncogene product of the Philadelphia chromosome, has been shown to be highly protective against hypoglycemia-induced apoptosis. These results demonstrate that PTK signaling can be either a positive or a negative regulator for the manifestation of hypoglycemia-induced survival in certain kinds of cancer cells.

## 5. Serum Deprivation

The cell culture condition termed serum starvation, as well as serum deprivation, depletion, removal, restriction, withdrawal, and serum limitation, has been widely and routinely used as a control point from which to examine a variety of extracellular stimuli or conditions (e.g., drugs, growth factors, hormones, and serum) [116]. However, given that cancer cells at the early stage often suffer from insufficient support by the local blood vessel, serum starvation by itself should be recognized as an important cellular condition through which to investigate cancer cell behavior. Many types of cancer and noncancer cell have been reported to adapt their growth and proliferation to serum-free culture conditions in a manner that depends on the release of growth factors or the modulation of cell surface receptors and/or intracellular kinases. Examples that have been demonstrated include growth factors such as EGF and other EGFR ligands [117–121], fibroblast growth factor-1 [122], PDGF [123], and VEGF [124, 125], PTKs such as c-Neu [126], ARK [127], and FAK [128], and serine/threonine kinases such as Akt [129–131], protein kinase C [132], GSK3*β* [133], adenosine monophosphate-dependent protein kinase [116, 131, 134], mammalian target of rapamycin [131], and Erk [130]. In many of these cases, protein phosphorylation is suggested to be responsible for suppression of cell death, namely, apoptosis. Thus, serum starvation provides an experimental system to analyze how normal cells undergo apoptosis in response to a shortage of mitogenic signals, and how malignant cancer cells escape the factors inducing apoptotic responses.

The roles of protein-tyrosine phosphorylation for the anti-apoptosis of cancer cells under serum-starved conditions have recently been fully documented by our studies on bladder carcinoma cell line 5637, whose serum-independent growth involving autocrine ligands for EGFR was reported in the 1990s [119–121]. We found that the activities of the two PTKs, namely Src and EGFR became stably upregulated after several hours of serum deprivation in culture medium [135]. The activated Src and EGFR contributed to the anti-apoptotic growth of 5637 cells under serum-starved conditions through phosphorylation of the *β*-subunit of c-Met/hepatocyte growth factor (HGF) receptor. In fact, inhibition of the Src/EGFR kinase activity or attenuation of the c-Met phosphorylation by knockdown (forced downregulation of c-Met by treatment with high doses of HGF) resulted in cell death accompanied by activation of caspase 3/7 and the appearance of apoptotic nuclear morphology [135]. In addition, it was demonstrated that cholesterol-dependent membrane microdomains (MDs) and their associated molecules, Src and uroplakin IIIa (UPIIIa), play important roles in signal transduction [136]. UPIIIa is a single transmembrane protein that has been originally identified as a major component of asymmetric unit membranes found in the luminal surface of mammalian urothelium [137, 138]. Its possible involvement in transmembrane (e.g. Src-dependent) signal transduction has also recently been demonstrated in uropathological bacterium infection [139] and in frog egg fertilization [140–143]. Thus, a novel signaling axis, involving MD and its associated proteins Src/EGFR/c-Met, has been identified as an anti-apoptotic mechanism that seems to be peculiar to bladder carcinoma cells. If so, further study should examine how other types of cancer cells become able to undergo malignant proliferation under serum-starved conditions.

## 6. Conclusion and Perspectives

**Figure 1:**
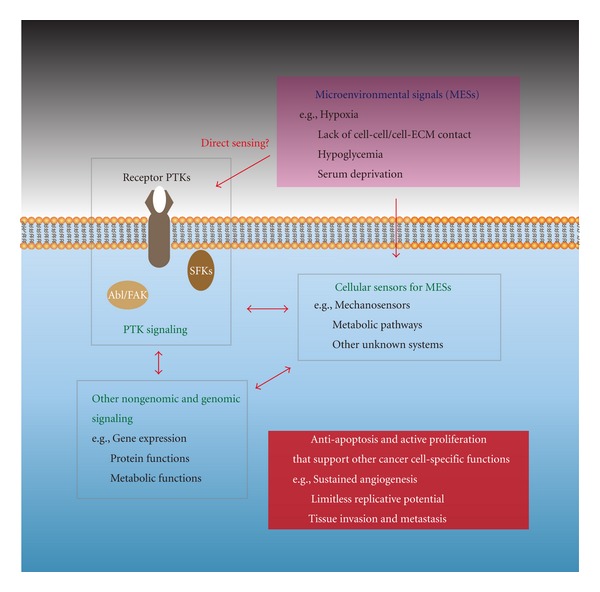
Signal transduction mechanism of anti-apoptosis in cancer cells. Highlighted here is the involvement of PTK signaling via nonreceptor PTKs such as SFKs, receptor PTKs such as EGFR, and cytoplasmic PTKs such as Abl and FAK in and around the plasma membranes. Several kinds of microenvironmental shortage for cell viability (e.g., hypoxia, lack of cell-cell or cell-ECM contact, hypoglycemia, deprivation of serum) act as signals (MESs, microenvironmental signals) for the responses of cancer cells (sensing of MESs, PTK signaling, other genomic and nongenomic signaling, and their crosstalk). These signaling networks support the cancer cells to undergo anti-apoptosis and active proliferation that lead to the other malignant features of cancer.

The ability to survive and continue active proliferation under stressful conditions described here (i.e., hypoxia, lack of anchorage, and low or no supply of glucose or serum) is one of the fundamental features associated with cancer cells of high malignancy. Many questions need to be answered about how cancer cells sense these micro-environmental signals (MESs) and undergo their adaptive behavior. Here, we have attempted to summarize the cutting edge views based on research dealing with the molecular mechanisms by which cancer cells evade the onset of apoptosis through the activation of Src and/or other PTK signaling, which occur mainly in and around the plasma membranes (Figure 1). On the other hand, accumulating evidence also demonstrates that such anti-apoptosis mechanisms also involve reversible protein phosphorylation on serine and threonine residues, and other posttranslational modifications of proteins and a number of metabolic pathways, which occur mainly in the cytoplasm or organelles such as mitochondria. In addition to the aforementioned nongenomic responses, genomic responses, that is, gene expression in the cell nucleus, also contribute substantially. Taking these findings together, further study in this research field should be directed towards learning how these arrays of knowledge could be integrated (Figure 1): for instance, understanding how the Src signaling pathway regulates its surrounding molecular network would contribute to understanding the mechanism of sensing extracellular/intracellular environments, the modulation of cellular metabolism, and the regulation of the genomic response and stability in cancer cell-specific functions.

## Abbreviations

RSV: Rous sarcoma virus
v-Src: Viral Src
c-Src: Cellular Src
PTK: Protein-tyrosine kinase
SFK: Src family kinase
SH: Src homology
VEGF: Vascular endothelial growth factor
pO_2_: Partial pressure of O_2_
HIF: Hypoxia-inducible factor
PI3K: Phosphoinositide (phosphatidylinositol) 3-kinase
Erk: Extracellular signal-regulated kinase
GSK3*β*: Glycogen synthase kinase 3*β*
FAK: Focal adhesion kinase
H/R: Hypoxia/reoxygenation
NF*κ*B: Nuclear factor kappa B
I*κ*B*α*: Inhibitor of NF*κ*B alpha
PTP: Protein-tyrosine phosphatase
LOX: Lysyl oxidase
ECM: Extracellular matrix
CAS: Crk-associated substrate
PDGFR: Platelet-derived growth factor receptor
EGFR: Epidermal growth factor receptor/kinase
JNK: c-Jun N-terminal kinase
HGF: Hepatocyte growth factor
MD: Membrane microdomain
UPIIIa: Uroplakin IIIa
MES: Micro-environmental signal.
